# Blastomycosis Awareness: A Crucial Reminder Amidst the Fifth Pneumonia Episode

**DOI:** 10.7759/cureus.75814

**Published:** 2024-12-16

**Authors:** Anam Umar, Allen Leland, Amber E Faquih, Beena Umar Ahsan

**Affiliations:** 1 Department of Internal Medicine, Ascension St. Vincent's Birmingham, Birmingham, USA; 2 Department of Infectious Disease, Ascension St. Vincent's Birmingham, Birmingham, USA; 3 Department of Infectious Diseases, University of Alabama at Birmingham, Birmingham, USA; 4 Department of Pathology, Henry Ford Health, Detroit, USA

**Keywords:** blastomyces dermatitidis, fungal lung infection, pulmonary blastomycosis, recurrent pneumonia, respiratory distress

## Abstract

Blastomycosis is a rare fungal infection endemic to North America and parts of Africa. It can be challenging to diagnose until it reaches a critical stage. We present a blastomycosis case in Alabama, emphasizing the importance of early recognition and management. A 67-year-old man had a month of worsening respiratory symptoms, which included malaise, cough, shortness of breath, and fever. Despite multiple urgent care visits and treatments with antibiotics and steroids, his condition continued to deteriorate. He was hospitalized with persistent fever and hypoxia. The imaging revealed patchy lung disease and multifocal consolidations, but initial cultures revealed no growth. He ultimately underwent bronchoscopy for budding yeast consistent with blastomycosis, along with influenza A. Treatment with amphotericin B was started, and there was a significant improvement. This case illustrates the need to consider the diagnosis of blastomycosis in a patient presenting with persistent respiratory symptoms unresponsive to conventional treatments.

## Introduction

*Blastomyces dermatitidis* is a dimorphic fungus commonly found in soil, particularly in specific regions of North America, including the southeastern, midwestern, and south-central United States, as well as the Ohio and Mississippi River valleys, northern Midwest, northern New York State, and southern Canada. When inhaled, the spores of this fungus can lead to blastomycosis, a potentially serious fungal infection. The incidence of blastomycosis in these endemic regions varies widely, ranging from 1.4 to 40 cases per 100,000 individuals [1].

Following inhalation, *B. dermatitidis* can cause an infection that may be asymptomatic or progress to acute or chronic pulmonary disease. In more severe cases, the infection can spread to other organs through the bloodstream, leading to significant illness and death. Prompt diagnosis and treatment are critical to preventing fatal outcomes associated with blastomycosis [2]. This case report highlights blastomycosis as a potential cause of recurrent pneumonia. Additionally, a literature review provides a comprehensive analysis of current research on the disease, covering its epidemiology, pathogenesis, clinical manifestations, diagnosis, and management.

## Case presentation

A 67-year-old man with a history of hypertension, asthma, and ulcerative colitis (UC) status postcolectomy in 2021, who was not on any immunosuppressants, presented to the emergency department (ED) with malaise, cough, shortness of breath, and subjective fevers that have persisted for about one month. Despite receiving oral antibiotics and steroids at an urgent care facility on at least five occasions, his symptoms failed to resolve. He had a similar illness in 2019, diagnosed as Candida pneumonia, which required prolonged therapy.

Clinical findings

Upon arrival at the ED, the patient was febrile and mildly hypoxic, with oxygen saturation between 91% and 94% on continuous pulse oximetry, requiring 2 L of supplemental oxygen via nasal cannula. Physical examination revealed mild wheezing without significant respiratory distress. The patient appeared fatigued and complained of generalized body aches.

**Figure 1:**
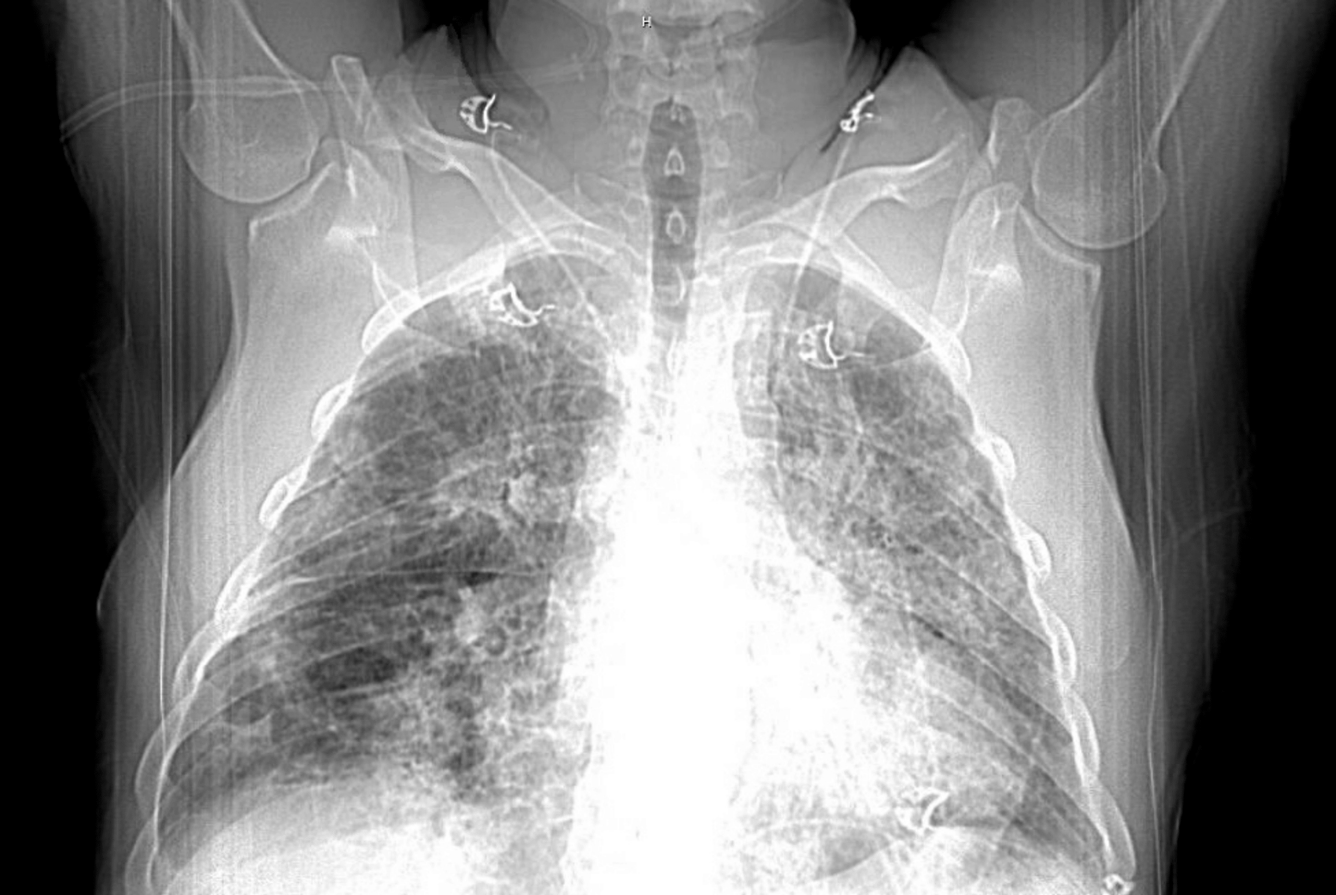
CT scan of the chest This image shows diffuse lung involvement with multifocal dense consolidations and scattered ground-glass opacities. There is a new cavitating lesion in the right lower lobe. The main pulmonary artery is dilated, and the mediastinal lymph nodes are prominent, likely due to a reactive process

**Figure 2:**
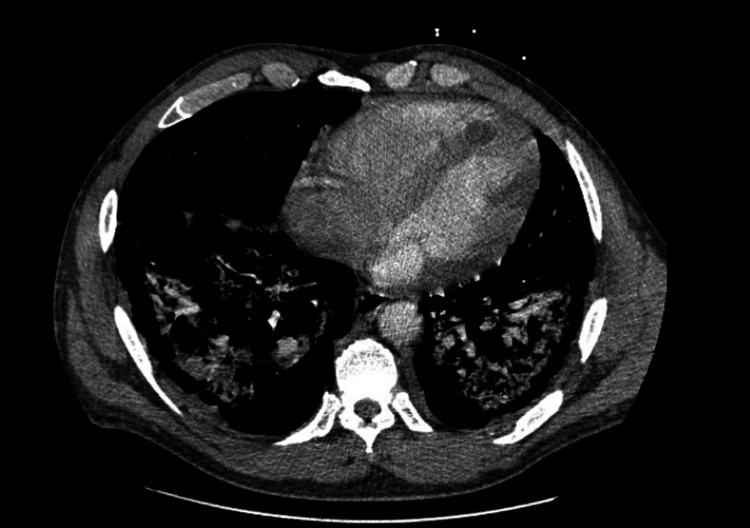
CT scan of the chest There is no CT evidence of pulmonary embolism in the proximal pulmonary arteries, although evaluation of the segmental and subsegmental arteries is limited due to bolus timing. Diffuse bilateral ground-glass opacities and consolidations have worsened since the previous exam. A dilated main pulmonary artery is observed, indicating pulmonary artery hypertension. Additionally, prominent mediastinal lymph nodes are likely reactive

**Figure 3:**
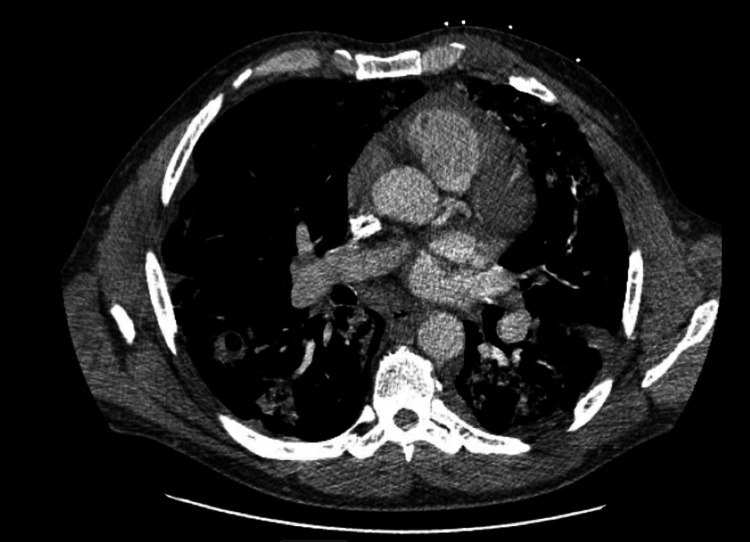
CT scan of the chest Due to the timing of the bolus, the evaluation of the pulmonary arteries is limited. No emboli were found in the central or lobar pulmonary arteries, and the main pulmonary artery is dilated. The scan showed no significant abnormality in the thoracic aorta or heart. There is no coronary artery calcification, but there are prominent reactive mediastinal lymph nodes. The lungs show worsening bilateral consolidations and ground-glass opacities, along with a cavitating lesion in the right lower lobe

A chest X-ray showed mild patchy right basilar airspace disease consistent with chronic obstructive pulmonary disease. In contrast, a chest CT scan revealed multifocal dense consolidations in the right upper, middle, and bilateral lower lobes, along with scattered ground-glass opacities, indicating diffuse lung involvement (Figures 1-3). No central airway obstruction was identified. Laboratory tests showed leukocytosis (white blood cell count of 16,000/μL) and acute kidney injury (creatinine level of 1.7 mg/dL). Empirical treatment with intravenous ceftriaxone and azithromycin for three days produced no improvement. Blood and sputum cultures showed no growth. Tests for COVID-19, influenza A and B, *Staphylococcus aureus*, respiratory syncytial virus, *Mycoplasma pneumoniae*, *Legionella pneumophila*, *Streptococcus pneumoniae*, aspergillosis, and HIV were all negative.

A consultation with the pulmonary service was requested due to the lack of response to conventional therapy and persistent symptoms suggesting an atypical or fungal etiology. A bronchoscopy with bronchoalveolar lavage was performed, and it revealed the presence of budding yeast, which was consistent with blastomycosis. Serological testing also confirmed influenza A, highlighting the multifactorial nature of the illness.

Treatment and follow-up

The patient was treated with liposomal amphotericin B of 5 mg/kg/day for one to two weeks as board spectrum antifungal, followed by oral itraconazole 200 mg twice daily for 12 months with close monitoring of clinical status, renal function, and response to therapy. The patient did not report a recurrence of symptoms during the follow-up and remained stable.

## Discussion

Blastomycosis is concentrated in five U.S. states: Arkansas, Louisiana, Michigan, Minnesota, and Wisconsin. It is not classified as a nationally notifiable disease, and limited surveillance contributes to an incomplete understanding of its prevalence in nonendemic areas [3]. In endemic regions, Blastomyces species thrive in sandy soils with acidic pH, decaying organic matter, forested areas, and rotting wood near water sources. Activities that disturb these soils, such as exploring beaver dams, construction, and outdoor recreation, are associated with outbreaks [4]. In nonendemic areas, cases are often linked to travel or latent infections in immunosuppressed individuals. While blastomycosis typically affects those from endemic areas who are immunocompromised, cases have also been reported in immunocompetent individuals and those from nonendemic regions [5,6]. Anderson et al. [7] documented delayed dissemination or reinfection with Blastomyces in immunocompetent hosts. Our patient, a known case of UC with a history of Candida pneumonia, had not been on immunosuppressants after colectomy in 2021. However, his immune system was likely compromised due to UC, an autoimmune condition leading to systemic inflammation, which can rarely affect the lungs. This impact on immunity and inflammation in inflammatory bowel disease (IBD) patients is discussed in a review by Ji et al. [8].

Furthermore, the literature suggests that in patients with IBD, the risk of opportunistic infections, including blastomycosis, extends beyond the use of immunosuppressive therapy. Age is an independent risk factor for these infections, with individuals over 50 showing a significantly higher prevalence of fungal infections, including aspergillosis, blastomycosis, candidiasis, and coccidioidomycosis [9]. A 2017 study on the epidemiology of opportunistic fungal infections in IBD patients from 2002 to 2014 found that 93% of blastomycosis cases in UC patients occurred in those over 50 years old. Among these cases, men accounted for 56.6%, and 92% of the patients were White. This increased prevalence is likely due to age-related immune decline, which predisposes individuals to opportunistic infections [10]. Although the use of immunosuppressants, such as biologics, increases infection risk, fungal infections like blastomycosis can also occur in nonimmunosuppressed UC patients, suggesting that factors such as comorbidities and age-related immune changes contribute to the risk. The increased mortality associated with these infections highlights the importance of early detection and management, particularly in older IBD patients. Geographic distribution, with significant cases in the Midwest and South, along with the predominance of large urban teaching hospitals, suggests regional factors may also influence diagnosis and outcomes [11]. In our patient, the primary risk factors for presenting with blastomycosis were UC and his age. Additionally, opportunistic fungal infections, such as candidiasis and herpesvirus infections, have also been observed in IBD patients even without immunosuppressive therapy. However, infections like histoplasmosis and mycobacterial infections were not specifically noted. This suggests that while immunosuppressive drugs significantly increase the risk of opportunistic infections, the underlying disease (IBD) may predispose patients to certain infections, albeit to a lesser extent [8].

Blastomycosis is transmitted through inhalation of soil spores, with an incubation period of 2-15 weeks [3]. Clinical presentations vary from asymptomatic to severe, including acute respiratory distress syndrome or extrapulmonary dissemination, depending on factors such as immune status, age, comorbidities (e.g., COPD and diabetes), and medications like steroids or immunosuppressants. Pulmonary involvement is most common (79%), often misdiagnosed as pneumonia, delaying treatment [12]. In immunocompromised patients, blastomycosis may become disseminated, with a mortality rate of up to 30%. Extrapulmonary manifestations include skin (40%-80%), bone (25%), genitourinary (10%-30%), and CNS (5%-10%) involvement, along with less frequent organ involvement such as the liver, spleen, lymph nodes, and adrenal glands [4]. Diagnosis involves the clinical history, physical exam, and laboratory tests, with potassium hydroxide preparations showing *B. dermatitidis* in respiratory secretions (sensitivity 36%-90%), but culture confirmation is necessary [13], and for suspected CNS involvement, cerebrospinal fluid analysis and MRI are recommended [14]. Early treatment is essential to slow the progression. Itraconazole is used for 6-12 months for mild-to-moderate pulmonary disease, while severe cases require amphotericin B for one to two weeks, followed by itraconazole. Extrapulmonary and osteoarticular infections also require prolonged treatment [15]. CNS involvement requires amphotericin B for four to six weeks, then voriconazole for at least one year [16]. Pregnant patients receive amphotericin B due to contraindications with azoles [17]. Therapeutic drug monitoring for itraconazole and voriconazole should occur after two weeks and 10 days of therapy, respectively [18]. Monitoring treatment adherence, adverse effects, and clinical response in the early stages is crucial for effective management and adjustments as needed.

## Conclusions

Blastomycosis can have various clinical symptoms influenced by the patient’s immune status. This case report stresses the need to consider blastomycosis in patients with recurrent pneumonia, regardless of geographic location or immune condition. Delayed diagnosis can worsen the disease. It is important not to treat pneumonia as just community-acquired pneumonia, especially in patients with a systemic illness history. Maintaining a broad differential diagnosis is essential.

In this case, it took five evaluations to reach the correct diagnosis, highlighting the importance of thoroughness in diagnosing to avoid missing less common causes of pneumonia. Healthcare professionals should be vigilant for blastomycosis to ensure timely diagnosis and treatment. Further research is needed to enhance our understanding of blastomycosis and improve early detection and management.
